# James A. Shapiro: Evolution: a view from the twenty-first century: FT Press Science, Upper Saddle River, New Jersey 07458, ISBN 10: 0-13278093-3

**DOI:** 10.1007/s12065-012-0069-4

**Published:** 2012-03-20

**Authors:** Paulien Hogeweg

**Affiliations:** Theoretical Biology and Bionformatics, Utrecht University, Utrecht, The Netherlands

Most readers of and contributers to this journal are likely to interpret “evolutionary intelligence” as the use of population based search techniques with random mutations and selection to generate ‘intelligent’ behavior. In contrast the bottom line of Shapiro’s book is (biological) evolution itself *IS* an ‘intelligent’ (‘cognitive’, ‘sentient’, ‘thoughtful’ are the words he uses) process. In the words of the first paragraph “life requires cognition at all levels” and in the concluding paragraphs: [21st view of evolution implies] “a shift from thinking about gradual selection of localized random changes to sudden genome structuring by sensory network-influenced cell systems…. It replaces the ‘invisible hands’ of geological time and natural selection with cognitive networks and cellular functions of self modification. The emphasizes is systemic rather than atomistic and information based rather than stochastic”.

Shapiro has been developing these ideas for a long time, initially inspired by Barbara McClintock’s classical work on chromosome repair and restructuring. He has recently advocated them in several papers (e.g. [1–3]) all mentioning that this is what 21st evolutionary theory is (will be) about.

The book builds up to these ideas through a very nice overview of current biological knowledge about information processing in cells. The review is extremely well documented (1,162 references in the book and an additional 50 pages of “extra references” online) and made more accessible to the general reader through an extensive glossary in the book (25 pages) and a list of recommended background reading on line (26 pages). Moreover it provides additional, more detailed information of some of the discussed processes online (together almost 100 pages, including (again) references).

In the first part of the book he reviews sensing and signaling mechanisms. He first explains the most classical example of metabolic regulation, namely the Lac operon, first described by Jacob and Monod in 1942, and the general information processing principles derived from it, often using computer derived terminology like “proteins operate as conditional microprocessors in regulatory circuits”. Then he focuses on regulatory processes involved in cell reproduction. He discusses first DNA repair and mutagenesis, showing that the latter can also be a regulated cellular process through error prone polymerases rather than externally imposed. Next the cell cycle and its checkpoints which ensure that different processes are coordinated. Then external signals, like pheromones, which regulate sexual vs clonal replication in yeast, and finally the machinery and regulation of cell death. This is all sound biology. Shapiro uses it to denounce the so called “central dogma of molecular biology”, i.e. ‘DNA makes RNA makes protein’, formulated by Crick in the early days of molecular biology (the beginning of the 70s).

In the second part “the genome as read-write storage system” he reviews ways in which DNA is modified. In my opinion this is the nicest part of the book and touches on biological processes which are least well known outside biology. He emphasises the larger scale modification, through e.g. transposons which make up the largest part of the DNA of higher eukaryotes like us. He describes the somatic recombination and editing which is employed in the immune system (in his words “to anticipate on future challenges”), and the recently discovered CRISPR system as ’immune’ system in bacteria, where parts of viruses are incorporated in the genome, and therewith helps to fight these viruses. Continuing to emphasis the capability of cells to modify DNA, he describes the startling reconstruction of the so called macro-nucleus from the micro-nucleus in Ciliates which involves extensive and precise DNA cutting and pasting and is guided by RNA’s. Finally, he explains the so called ’epigenetic’ modification of DNA e.g by methylation which renders parts inaccessible, not only on the short term but also over several generations. This again is sound and interesting biology. He concludes his overview of DNA modification as follows: “one of the most profound lessons from the past 6 decades of molecular cell biology that all aspects of cell functioning and cell biochemistry are subject to regulation. We have no scientific basis for postulating that genome functioning is different in this regard. In other words we have every reason to expect that natural genetic engineering is also subject to regulation and do not operate in an uncontrolled way…” and continues to list a number of cases where e.g. starvation, antibiotics or DNA damage trigger various forms of DNA modification or transposon activation. He stresses that he thinks that the known regulatory processes are only the tip of the iceberg, and that much remains to be discovered.

In the third section he reviews some insights obtained from the large genome sequencing projects, emphasizing the dynamics of genomes, rewiring of regulation, novelty through duplications (and deletions) of larger stretches of the genomes, horizontal gene transfer and whole genome duplication at critical geological transition, etc. He presents these as evidence for his concept “natural engineering”; in the terminology of artificial evolution they might be called a rich set of genetic operators. As an example of his natural engineering claim he mentions that the difference of mouse and man is in the different repertoires of certain repeats (SINE elements) and concludes that “although we are largely ignorant of how they organize large scale physiological organization, they do contain recognizable signals of regulatory elements”. The book has many such suggestive sentences, which however fall far short of substantiating his far reaching claims.

Why don’t I like the book as much as much I would like to like it? First, why I would like to like it. I do agree that evolutionary theory has been slow to incorporate the current knowledge on cellular information processing. I also fully agree that to do so will have a profound impact and deepen and enrich our understanding of biological evolution and through understanding evolution of “why organisms are what they are”. In addition it could lead to better applications of evolutionary theory as pursued in this journal.

However, unfortunately, Shapiro tends to grossly oversell his case, which I find irritating. Calling evolution (and cells) ‘cognitive’, ‘sentient’ and ‘thoughtful’, is in my opinion not very illuminating, nor does it set a clear research agenda. As is apparent from the above quotes, although the biology he presents is solid, his conclusions are only loosely connected to it via suggestive language. Also, he tends to exaggerate difference with earlier (sometimes long outdated) ideas at the cost of not only historical but also factual accuracy. For example, denouncing Crick’s “DNA makes RNA makes protein” he reverses it in “protein + ncRNA + signals + other molecules + structures − > phenotype + genotype (+epigenotype)”. Certainly there should be a circle in there somewhere.

Nevertheless, the biology reviewed in the book poses two interesting challenges for “in silico evolution” (I use this term in silico evolution not to hit on any in my eyes spurious subdivisions made in the field). First, is it sensible to include any of the regulatory processes of modifying genetic material in our algorithms, and if so how can we do it and how can it increase the power of the evolutionary process (for whatever application it is used).

And secondly the, in my view more profound, challenge is to unravel how evolution generated such richly structured, versatile evolving systems that biological experiments have shown biological systems to be. This question is conspicuously absent throughout the book, and he explicitly claims in the last chapter that it is(presently) outside the realm of science. In my opinion in silico evolution however can shed light on the evolution of evolution if it is allowed enough degrees of freedom. Indeed this is currently my main research focus, the main results relevant to the present discussion are reviewed in [4]. Taking the classical Darwinian random mutation and selection as starting point, we have shown that through genome structuring through transposable elements, and through evolved genotype phenotype mapping, long term evolution leads to random mutations which are non-random in occurrence and/or effect and biased to advantageous mutations. We have also shown that the genome dynamics, gleaned from phylogenetic reconstruction, and experimental evolution, is mimicked in our models. This is just a beginning, but it shows we are still far from understanding what the basic paradigm of “random mutation selection” can do. Much remains to be discovered (yes in the twenty-first century!).
